# *Salvia officinalis* L.: Antitrypanosomal Activity and Active Constituents against *Trypanosoma brucei rhodesiense*

**DOI:** 10.3390/molecules26113226

**Published:** 2021-05-27

**Authors:** Núria Llurba Montesino, Marcel Kaiser, Pascal Mäser, Thomas J. Schmidt

**Affiliations:** 1 Institute of Pharmaceutical Biology and Phytochemistry (IPBP), University of Münster, PharmaCampus, Corrensstr. 48, D-48149 Münster, Germany; nunu.llum@gmail.com; 2 Swiss Tropical and Public Health Institute (Swiss TPH), Socinstrasse 57, CH-4051 Basel, Switzerland; marcel.kaiser@unibas.ch (M.K.); pascal.maeser@swisstph.ch (P.M.); 3 University of Basel, Petersplatz 1, CH-4003 Basel, Switzerland

**Keywords:** *Salvia officinalis* L., *Trypanosoma brucei rhodesiense*, antitrypanosomal activity, diterpene, abietane, rosmanol derivative

## Abstract

As part of our studies on antiprotozoal activity of approved herbal medicinal products, we previously found that a commercial tincture from *Salvia officinalis* L. (common Sage, Lamiaceae) possesses high activity against *Trypanosoma brucei rhodesiense (Tbr)*, causative agent of East African Human Trypanosomiasis. We have now investigated in detail the antitrypanosomal constituents of this preparation. A variety of fractions were tested for antitrypanosomal activity and analyzed by UHPLC/+ESI QqTOF MS. The resulting data were used to generate a partial least squares (PLS) regression model that highlighted eight particular constituents that were likely to account for the major part of the bioactivity. These compounds were then purified and identified and their activity against the pathogen tested. All identified compounds (one flavonoid and eight diterpenes) displayed significant activity against *Tbr*, in some cases higher than that of the total tincture. From the overall results, it can be concluded that the antitrypanosomal activity of *S. officinalis* L. is, for the major part, caused by abietane-type diterpenes of the rosmanol/rosmaquinone group.

## 1. Introduction

Human African Typanosomiasis (HAT or “sleeping sickness”) is one of 20 diseases currently classified by WHO as neglected tropical diseases (NTDs). HAT, as well as the cattle disease Nagana, are caused by the kinetoplastid parasite *Trypanosoma brucei* subsp. Ref. [1] and are usually lethal if untreated. The few existing medications for HAT are toxic or difficult to administer. Drugs in use against Nagana suffer from increasing parasite resistance. The identification of new active chemical entities against *T. brucei* is, therefore, an urgent need. Recently, the first orally active trypanocidal drug to treat late-stage HAT, fexinidazole [2], was introduced. However, it remains important to identify further new active compounds, against the diseases affecting humans as well as animals, possibly with new mechanisms of action, in order to develop further drugs against African trypanosomes.

In a previous communication, we reported on the antiprotozoal activity of 53 herbal medicinal products (HMPs) approved in Germany and commonly marketed for other ailments. In that screening, an alcoholic tincture of *Salvia officinalis* L. (common Sage, Lamiaceae) exhibited strong and selective *in vitro* activity against *T. brucei rhodesiense*, the etiologic agent of East African HAT, with an IC_50_ value of 1.9 µg/mL and a selectivity index of 17 [3]. These results appeared promising and various other *Salvia* species previously showed antiprotozoal activity [4], so that a detailed study of the active principles in *S. officinalis* L. tincture was initiated. In the present study, we report on the identification of the main antitrypanosomal constituents from this HMP, using a multivariate statistics-guided isolation approach, and their bioactivity against the pathogen.

## 2. Results and Discussion

### 2.1. Fractionation of Sage Tincture and Antitrypanosomal Activity of the Resulting Fractions

The Sage extract, obtained after evaporation of the alcoholic tincture to dryness, was separated by CC on Sephadex ® LH-20 with an isocratic mobile phase of MeOH:H_2_O (80:20 *v*/*v*), affording 38 fractions (FR1-FR38).

**Figure 1:**
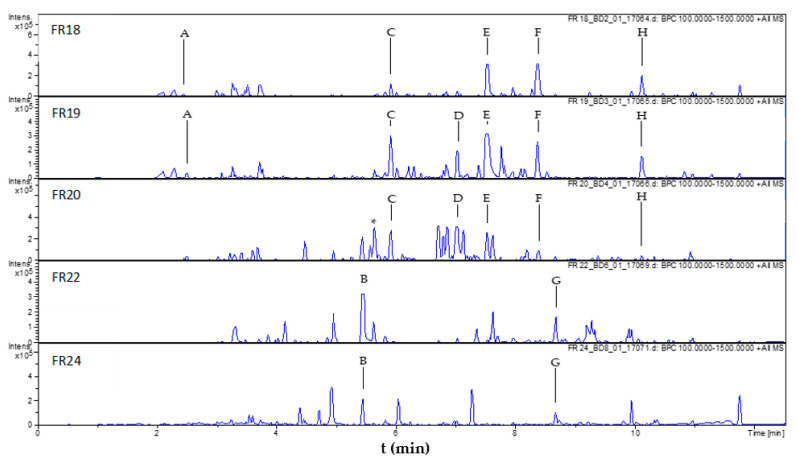
LC/MS profiles (base peak chromatograms of *m*/*z* 100–1500) of the most active fractions obtained from Sage tincture. Letters (A–H) denote the eight constituents predicted by the PLS model to be of major impact on bioactivity. * Compound **3**, not highlighted by the PLS model but isolated.

Samples with a defined concentration of 2 mg/mL were prepared of each fraction and analyzed by UHPLC/ESI-QqTOF-MS (henceforth abbreviated LC/MS) to determine the analytical fingerprints. After the analysis and dereplication of major peaks (see Figure S1 and Table S1 in Supplementary Material) as well as comparison of the samples’ analytical profiles, 17 representative fractions were selected to be assayed in vitro against *Tbr*. The growth inhibition (GI) results of the fractions are reported in Table 1 and Figure 1). Ten of the tested fractions exhibited more than 50% GI at 10 µg/mL, five of which (FR18, FR19, FR20, FR22, and FR24) yielded high activity > 75% GI, FR19 and FR20 being the most active with 100% GI. IC_50_ values for the individual fractions were not determined, in order to save resources and since the % GI data can serve as an approximate substitute in cases where relative activities of similar samples such as the fractions under study are compared. A comparison of the LC/MS fingerprints of the most active fractions is presented in Figure 1.

### 2.2. Partial Least Squares (PLS) Regression Model to Predict Constituents with Main Impact on Bioactivity

The LC/MS data of all tested fractions were processed and transformed using Profile Analysis v2.0 (Bruker Daltonik GmbH) to obtain the data matrix (X-matrix) of independent variables or predictors. This matrix consisted of 230 X-variables represented by tR:*m*/*z* pairs with their respective signal intensity for each of the 17 fractions (observations). This matrix was then exported to The Unscrambler v. 9.2 (Camo Process AS, Oslo, Norway, 2005) and used to calculate a PLS model with the % GI values at 10 µg/mL as Y-variable. The 10 µg/mL data were chosen since they presented a much higher variance than those obtained with 2 µg/mL.

**Figure 2:**
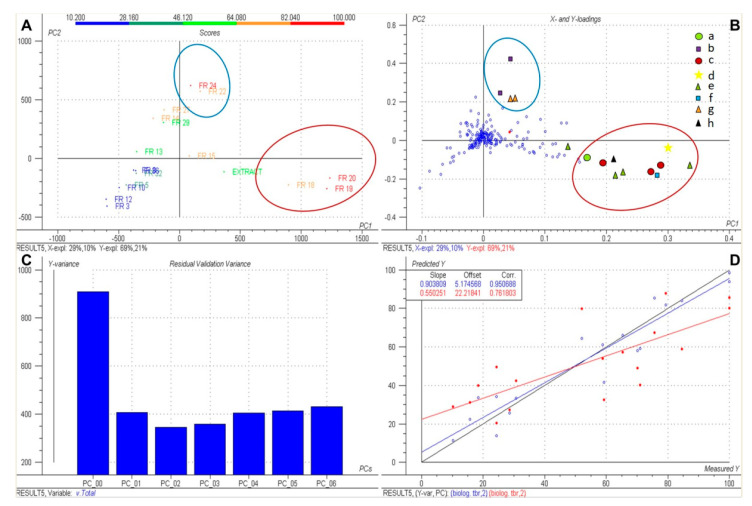
Overview of the PLS model. (**A**) Scores plot of the second vs. the first PLS component; (**B**) loadings plot corresponding to (**A**); (**C**) residual variance plot; (**D**) plot of predicted vs. measured GI (%) data. Blue dots: model calibration. Red dots: leave-one-out cross validation. In (**B**), the variables representing peaks A–H are highlighted with different symbols.

The PLS model thus obtained explains about 90% of the variance in the Y-variable (biological data) with two PLS components and shows a reasonable internal predictivity as assessed by leave-one-out cross validation (Figure 2). The scores plot (Figure 2A) of the second versus the first PLS component (PC2 vs. PC1) shows the positions of each fraction in the two-dimensional latent variable space defined by the second plotted vs. the first PLS component. The fractions were colored according to their bioactivity. FR18, FR19, and FR20 are situated close to each other with high scores on the first PLS component but far from FR24 and FR22, which are close to each other with high scores on the second component. This indicates that the compound(s) responsible for the activity of FR24 and FR22 are different from those responsible for the high activity of FR18, FR19, and FR20. The loadings plot (Figure 2B) defines the relation among the original (measured) variables and the latent variables (PLS components), i.e., the influence of each individual X-variable on the model. The position of the variables in the loadings plot is thus correlated with the fractions’ position in the scores plot (Figure 2A), which facilitates the localization of the most important constituents (highlighted with blue and red circles, respectively) by investigating the LC/MS data in the respective fractions. Inspection of the loadings plot (Figure 2B) thus allowed us to highlight eight constituents (A–H) with high loadings on the first and second PLS components, i.e., strongest influence on biological activity against Tbr. (Note that, in most cases, more than one tR:*m*/*z* variable represents the same compound). These constituents were localized in the LC/MS analyses. The high-resolution mass spectral data allowed assignment of presumable elemental compositions of those relevant constituents (see Table 2).

The correlation plot (Figure 2D), which represents predicted versus experimental growth inhibition (%) data of fractions given by the PLS model, indicates a good prediction of the PLS model with a correlation coefficient R of 0.95 (i.e., explained variance, R^2^ of 90%; blue dots) and a correlation of leave-one-out cross validation predictions (red dots) of 0.76 (i.e., Q^2^ = 0.58).

The LC/MS peaks of the targeted compounds highlighted by the PLS models are marked in Figure 1 and their mass spectral characteristics are summarized in Table 2.

Based on these results, the isolation of the compounds with the most important contributions to activity according to the PLS models was carried out.

### 2.3. Identification of the Compounds Selected by the PLS Model

The constituent represented by peak A (see Figure 1) was the only one that could neither be dereplicated on the grounds of its MS data nor isolated, due to its very low content in the Salvia extract and FR18 and FR19. Since it did not present a very high loading in the model, no further attempts were made to identify this constituent.

**Figure 3:**
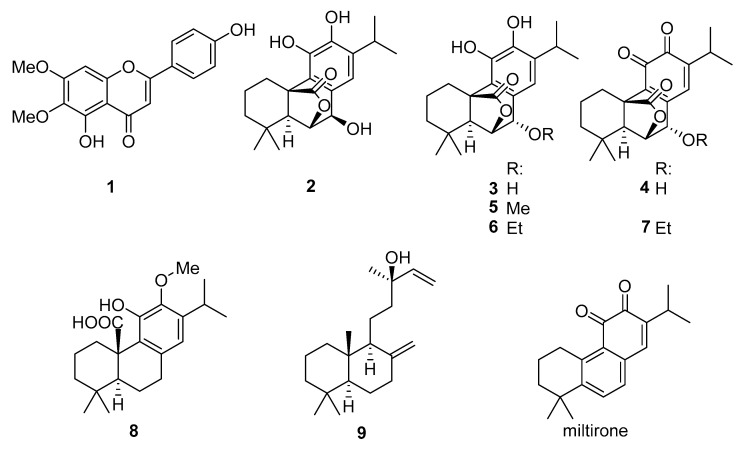
Structures of the isolated compounds (**1**–**9**) and miltirone.

Peak B (Figure 1, tR = 5.5 min) was dereplicated by comparison of its MS data with those of known constituents of *S. officinalis* L. A constituent of fractions FR22 and FR24, it was identified as the dimethoxy flavone cirsimaritin (4’,5-dihydroxy-6,7-dimethoxyflavone, [M+H]^+^ at *m*/*z* 315.0864, calcd 315.0863: C_17_H_15_O_6_^+^, [M+Na]^+^ at *m*/*z* 337.0682, calcd 337.0683: C_17_H_14_O_6_Na^+^; compound **1**, see Figure 3). A sample of authentic cirsimaritin was available from previous research, where the compound had also been found to show antitrypanosomal activity [5] so that it did not have to be isolated. The retention time and mass spectral data of the reference compound were identical with those of peak B.

Peak C (Figure 1 (tR = 5.9 min) was suggested by its LC-MS data ([M+H]^+^ = 347.1879, calcd 347.1853 C_20_H_27_O_5_^+^) to be the diterpene rosmanol, or an isomer thereof. The compound was isolated from fraction FR20 (2.2 mg) and found by analysis of its NMR data in comparison with literature [6] to be epirosmanol (**2**). It is to be noted that another compound of the same elemental composition, which had not been selected by the PLS model, was also isolated from the same fraction (13 mg; peak denoted with an * in Figure 1; tR = 5.7 min, [M+H]^+^ = 347.1847; compound **3**) and found to be identical to rosmanol by its NMR data [6]. The latter was accompanied by the corresponding o-quinone (rosmaquinone **4**, a probable oxidation product of **3**) with which it formed a mixture of approximately 4:1; **4** was also unambiguously identified by its NMR data in comparison with literature [7]. Compounds **2**, **3,** and **4** (see Figure 3) have been reported as known constituents of *S. officinalis* L. [8].

From fraction FR20, peak D (Figure 1, tR = 7.0 min, [M+H]^+^ at *m*/*z* 361.2010; calcd 361.2010: C_21_H_29_O_5_^+^) was isolated (8 mg). It could unambiguously be identified by its NMR data [9] as the methyl ether of **3**, i.e., 7-*O*-methylrosmanol (**5**).

Likewise, peak E (tR = 7.5 min, [M+H]^+^ at *m*/*z* 375.2158 calcd 375.2166: C_22_H_31_O_5_^+^), after isolation from fraction FR19 (3 mg), was found to be the corresponding ethyl ether of **3** (i.e., 7-*O*-ethylrosmanol, **6**), a known constituent of *S. officinalis* L. [10]. As in the case of **3**, **6** was accompanied by the corresponding o-quinone (i.e., 7-*O*-ethylrosmaquinone, **7**; [M+H]^+^ at *m*/*z* 373.2004 calcd 373.2010: C_22_H_29_O_5_^+^). Both were unambiguously identified by their NMR data, which were in agreement with those in the literature [6,11]. Compounds **6** and **7** were obtained as a mixture with a further, unrelated, impurity in a ratio of approximately 6:4:1.

The compound underlying peak F, present in fractions 18–20, was suggested by its MS data to be 12-*O*-methylcarnosic acid (**8**), ([M+H]^+^ at *m*/*z* 347.2205, calcd 347.2217: C_21_H_31_O_4_^+^). An authentic sample of 12-*O*-methylcarnosic acid was available so that compound **8** could be directly compared with the reference compound by LC/MS and, thus, did not have to be isolated. The retention time and mass spectrum were found identical so that it was unambiguously identified as this diterpene, a known constituent of several Salvia species [12,13].

The constituent yielding peak G, present in fractions 20–24, according to the LC-MS dereplication results from fraction FR22 (tR = 8.7; [M+H]^+^ at *m*/*z* 283.1702, calcd 283.1693: C_19_H_23_O_2_^+^), was expected to be identical with miltirone, a diterpene known from S. miltiorrhiza, but not previously isolated from *S. officinalis* L. Miltirone was previously reported to possess very potent activity against Tbr [14]. Due to the low concentration in the investigated material, a successful isolation turned out to be impossible. An authentic sample of miltirone was obtained, but the identity was disproven by direct co-chromatography (UHPLC/ESI-QqTOF MS/MS), where the retention time differed significantly. Peak G must, therefore, represent an isomer of miltirone (hitherto unknown in *S. officinalis* L., to the best of our knowledge), which, at present, must remain uncharacterized. It is to be noted that this constituent occurred only in very small quantities and also displayed only relatively small loading values on the second component of the PLS model, so that its contribution to the overall activity of Sage tincture is probably not very strong.

Compound **9**, represented by peak H, was not isolated from the fractions in which it occurred during the profiling (FR18–FR20) but was obtained from a hexane extract prepared by solvent partitioning. By NMR, it was identified as the common labdane diterpene manool [15,16], which is widespread in plants and also known as a constituent of Salvia species [16,17] (see Figure 3). It turned out that, in this case, the [M+H]^+^ ion was not detected by LC/+ESI MS under the conditions applied here, but that the ion occurring at the highest *m*/*z* value (273.2587) represents the fragment formed from the former by loss of a molecule of water ([M+H−H_2_O]^+^, calcd 273.2577). When analyzed by GC/EI MS, the molecular ion [M]^+^ at *m*/*z* 290 was very low in abundance, but detectable.

### 2.4. Antitrypanosomal Activity of the Isolated Compounds

After isolation and identification, the biological activity of compounds **1**–**9** against Tbr was tested in vitro. Melarsoprol was used as positive control. The cytotoxicity of the compounds was assessed using L6 rat skeletal myoblast cells and podophyllotoxin as a reference drug. The IC_50_ values are reported in Table 3.

As mentioned above, Tbr exhibited high sensitivity toward the total extract obtained from Sage tincture (IC_50_ = 1.86 µg/mL and SI of 17). The range of biological activity of the isolated compounds was from 1.0 to 15 µg/mL, so that some compounds (**1**) and mixtures (**3**+**4** and **6**+**7**) displayed higher activity than the total extract. Of these, compounds **3** and **4** were not selected by the PLS model but, nevertheless, showed interesting activity (IC_50_ = 1.1 µg/mL and SI of 4.9). However, none of the tested substances displayed exceptionally high activity, so that the overall activity was probably a sum of their activities (and, possibly, similar compounds present in minor amounts).

The activity of the flanovonoid **1** against Tbr was previously reported. The IC_50_ value of the present study was only somewhat lower than that of the previous study (1.7 vs. 3.3 µg/mL [5]). The methoxylated carnosic acid derivative **8**, which had previously shown more promising activity against Leishmania donovani [13], displayed activity against Tbr at a rather low level.

The various diterpenoids with the common rosmanol skeleton (compounds **2**–**7**) displayed more promising antitrypanosomal activity, though to variable extent. Thus, the mixtures of **3** with **4** (rosmanol + rosmaquinone) and **6** with **7** (7-*O*-ethylrosmanol+ 7-*O*-ethylrosmaquinone) were the most active samples with IC_50_ values of 1.0 and 1.1 µg/mL, respectively, whereas compound **3** (7-*O*-methylrosmanol) was the least active compound of this group with an IC_50_ of about 8.3 µg/mL.

It is tempting to hypothesize that the low activity of **3** was related with the catechol moiety being protected by methoxylation. The observation that the mixtures of compounds **3** and **6** with their corresponding quinones, **4** and **7**, respectively, yielded the highest activity, whereas **2**—not accompanied by its quinone form at the time of analysis—was somewhat less active, which might indicate that higher antitrypanosomal activity among these diterpenoids was conferred by the quinones rather than the catechols. It will be interesting to investigate this after isolation of larger quantities of the respective diterpenes in a following study.

Interestingly, the tR:mz variables representing peak B (compound **1**) and, to a lesser extent, also peak G (a putative isomer of miltirone) were present in the later fractions (FR22, FR24), while those of the other diterpenes C–F and H abounded in the earlier fractions (FR17–FR20). This was reflected in the PLS model, where these two groups contributed to the different latent variables, PC2 and PC1, respectively, in Figure 2A,B. The first component accounting for a much higher fraction of the explained variance in the biological response data (69 vs. 21%, see Figure 2A,B), it can be expected that the compounds with high loadings on PC1 were also more important for the observed differences in activity between the fractions. The compounds underlying peaks C–F (i.e., **2**, **5**, **6**–**8**) were present in the total extract in much higher quantities than B and G, so that it may safely be assumed that they accounted for the major part of the overall antitrypanosomal effect of Sage tincture. It is worth noting that, in spite of various reports on antiprotozoal activity of Salvia diterpenes from other species (see [4]), no previous reports exist on such activity for rosmanol-type compounds such as the ones reported here. Further studies on such diterpenes have been initiated.

## 3. Materials and Methods

### 3.1. Investigated Materials

The investigated Sage tincture was the same commercial product as investigated in our previous communication [3]. A reference sample of 12-*O*-methylcarnosic acid (**8**) was provided by Phytolab GmbH & Co KG, Vestenbergrsgreuth, Germany, and a sample of authentic miltirone was provided by Dr. S. Ślusarczyk, Wroclaw Medical University, Wrocław, Poland.

### 3.2. General Methods

Column chromatography (CC) was performed at ambient pressure and room temperature using Sephadex LH-20 or Silica 60 as stationary phase in glass columns of appropriate size.

Analytical thin-layer chromatography (TLC) was performed on silica 60 F 254 gel-coated aluminum sheets (20 × 20 cm; Merck, Darmstadt, Germany), usually developed over a distance of 8 cm and then observed under UV light (254 nm) followed by spraying with Anisaldehyde reagent (MeOH: acetic acid: sulphuric acid (85:10:5) + 0.5 mL Anisaldehyde (4-Methoxybenzaldehyde). For analytical purposes, the sheets were cut into two 10-cm halves. The samples were applied in bands (width of 1 cm) and the plates developed over a distance of 8 cm with the eluent mixtures mentioned below.

Preparative HPLC separations were performed on a Jasco (Gross-Umstadt, Germany) HPLC system consisting of a PU 2087 Plus Pump with DG2080-54 degasser, a AS2055 autosampler with 2000-µL injection loop and a C = 2060 column thermostat. The column used for the separations described below was a Nucleodur 250/10 C18 Htec 5 µm (Macherey-Nagel, Düren, Germany).

All NMR measurements were carried out at the Institute für Pharmazeutische und Medizinische Chemie der Universität Münster under the supervision and responsibility of Dr. Jens Köhler and staff. One- and two-dimensional NMR assays (1H, 13C, HMBC, HSQC, COSY, NOESY) were recorded on Agilent DD2 400 MHz or DD2 600 MHz spectrometers (Agilent Technologies, Santa Clara, CA, USA). The measuring frequencies are indicated for each compound in the respective table of NMR results. NMR spectra were acquired in CDCl_3_ or DMSO-d_6_ (see NMR tables in Supplementary Materials) at room temperature and processed with the software MestReNova, vs. 11.0. Spectra (Mestrelab Research, Chemistry Software Solutions, Santiago de Compostela, Spain). The spectra were referenced to the CDCl_3_ or DMSO-d_6_ solvent signals (1H, 7.260 ppm and 2.500 ppm/3.330 ppm, respectively). Chemical shifts (δ) in ppm, the peak integral size, the multiplicity, and coupling constants (J) in Hz were analyzed for every sample. For ^13^C NMR solvent signals (13C, 77.000 ppm and 39.520 ppm, respectively), the chemical shifts (δ) in ppm were analyzed.

### 3.3. Extraction and Isolation

The dried extract (5.1 g) obtained by evaporation of 300 mL of Sage tincture under reduced pressure was resuspended in 33 mL of MeOH-H_2_O (8:3, *v*/*v*), passed through a cotton filter, and subjected to CC on Sephadex ® LH-20 (400 g) packed and eluted isocratically with the same solvent system at a flow rate of 1 mL/min. The eluates were collected in portions of 15 mL. Eluates were collected and controlled by TLC (EtOAC:MeOH:H_2_O 100:13.3:10, visualized under UV and by derivatization with anisaldehyde spray reagent). They were combined according to their TLC profiles into 38 fractions (FR1–FR38).

FR20 (230 mg) was further separated into four portions of 50–60 mg by CC on 5g Europrep C18-RP 20–45 µm (4.5 × 1.5 cm), with a gradient of H_2_O (A): MeOH (B) with 50% B: 10 mL; 75% B: 10 mL; 100% B: 20 mL; re-conditioning with 10 mL 50% B. The eluates were combined according to their TLC profiles into nine subfractions (FR20-1–FR20-9). FR20-1–FR20-3 were eluted with 50% B, FR4–FR6 were eluted with 75% B, and FR20-7 and FR20-8 were eluted with 100% B, while FR20-9 was collected during re-equilibration.

From FR20-5 (58 mg), compound **2** (2 mg, tR = 11.8) and a mixture of compounds **3**+**4** (13 mg, tR = 17.2 min) were isolated by semiprep. HPLC was done, using the following gradient of H_2_O (A): MeOH (B) with 65% B (0-9 min); 65–80% B (9–11 min); 80–100% B (12.2–20 min); 100–65% B (20–22 min); and re-conditioning was with 65% B from 22–26 min. Flow rate was 2.8 mL/min and column temperature was 40 °C.

FR20-6 (29 mg) was separated by semiprep. HPLC was with a gradient of H_2_O (A): MeOH (B) with 70% B (0–2.1 min); 70–80% B (2.1–4.1 min); 80–100% B (9.1–11.1 min); 100–70% B (15.2–18 min); and re-conditioning was with 65% B from 18–22 min. Flow rate was 2.8 mL/min, 30 °C, to yield 8 mg of compound **5**, eluted with tR = 14.0 min under these conditions.

From FR19 (39 mg), the mixture of compounds **6**+**7** (tR = 17 min) was isolated by semiprep. HPLC was done using a gradient of H_2_O (A):MeOH (B) with 50–80% B (0–4 min); 80–100% B (4–10 min); 100–30% B (12.2–20 min), 30–50% B (20–24 min); and re-equilibration with 20% B from 24–25 min. Flow rate was 2.8 mL/min, 40 °C.

Compound **9** was isolated from a separate workup of Sage tincture, in which 200 mL of the tincture were evaporated. The residue (3.4 g) was suspended in MeOH:H_2_O (2:3) and partitioned with 3 × 300 mL n-hexane. The residue after evaporation of the hexane partition (0.38 g) was subjected to CC on 150g, Silica 60 eluted isocratically with 100% n-hexane. Eluates were controlled by TLC and visualized under UV and by derivatization with anisaldehyde spray reagent. They were combined, according to the TLC profiles, into 37 fractions Hex1-Hex37. Fraction Hex6 represented a single constituent, compound **9** (3 mg).

*Analytical data:* +ESI QqTOF MS see Table 2. NMR spectra and data are presented as supplementary material (Figures S2–S11 and Tables S2–S6, supplementary material).

### 3.4. UHPLC/+ESI QTOF MS and MSMS Analysis

All analyses were performed on a Bruker Daltonics (Bremen, Germany) micrOTOF QII quadrupole/time-of-flight mass spectrometer with an Apollo electrospray ion source operated in positive ionization mode and coupled to a Dionex Ultimate 3000 RS Liquid Chromatography System (Idstein, Germany) with a Dionex Acclaim RSLC 120, C18 column (2.1 × 100 mm, 2.2 µm, protected by an EXP guard column 3 × 5 mm, C-18, 1.8 µm) using a binary gradient of water and acetonitrile, both with 0.1% formic acid. The LC system was additionally equipped with a Dionex Ultimate DAD-3000 RS (wavelength range of 200–400 nm) UV/Vis detector.

The samples were dissolved in methanol at a concentration of 2 mg/mL. The injection volume was 10 µL. Separations were performed using a binary gradient of A: water, B: acetonitrile, both with 0.1% formic acid at a flow rate of 0.4 mL/min: from 0 to 0.2 min, isocratic at 5% B; 0.2 to 9.7 min, linear from 5% B to 100% B; 9.5 to 12.5 min, isocratic at 100% B; 12.5 to 12.6 min. linear from 100% B to 5% B; 12.6 to 15 min, isocratic at 5% B. The column was thermostatted to 40 °C. Eluted compounds were detected in UV/Vis over a wavelength range of 200–400 nm and by QTOF mass spectrometry in positive mode at 4 Hz over a mass range of *m*/*z* 100–1000 using the following instrument settings: nebulizer gas nitrogen, 4 bar; dry gas nitrogen, 9 L/min, 220 °C; capillary voltage, 4500 V; end plate offset, −500 V; transfer time, 100 µs; collision gas nitrogen. Collision energy and collision RF (Radio Frequency) settings were combined to each single spectrum of 1250 summations as follows: 624 summations with 80 eV collision energy and 130 Vpp + 313 summations with 16 eV collision energy and 130 Vpp + 313 summations with 16 eV collision energy and 130 Vpp. Internal data set calibration (HPC (High Precision Calibration) mode) was performed for each analysis using the mass spectrum of a 10 mM solution of sodium formiate in 50% isopropanol that was infused during LC re-equilibration using a divert valve equipped with a 20-µL sample loop.

### 3.5. Data Treatment and PLS Modeling

The X matrix was built from the LC/MS analyses of the 17 fractions with determined IC_50_ values, all performed in one coherent series, calibrated, processed, and converted to a descriptor table (“bucket table”) using the software ProfileAnalysis (Version 2.0, Bruker Daltonik GmbH), applying the following parameters: Buckets were generated within a retention time range from 2 to 12 min and an *m*/*z* range of 100 to 800. Using the advanced bucketing function, individual buckets were built with a time window of 0.2 min and an *m*/*z* window of 25 mDa. They were only considered if present in at least three of the 17 analyses. The signal/noise threshold for recognition of molecular features was set to 5 with a correlation coefficient of 0.7 and a minimum compound length of 10 spectra. A smoothing width of 2 was applied. This resulted in a total of 230 bucket variables of the form <tR(min):*m*/*z*>, which were transformed to square root values for further analysis. The descriptor matrix (X-matrix, independent variables) thus consisted of N = 17 observations x K = 230 descriptors. The table was exported to Microsoft Excel and the biological activity data expressed as the fractions‘ percentage of growth inhibition against Tbr at 10 µg/mL were added to constitute the Y matrix (dependent variable). The complete data table was then imported to The Unscrambler v 9.2 (Camo Process AS, Oslo, Norway), which was used to calculate the PLS regression model. The variable data were used without normalization or scaling since it was found that this procedure yielded the best model in terms of statistical performance (correlation, cross validation). It appeared that normalization was not necessary since all samples were analyzed in identical concentration, under identical conditions, and on the same day. Scaling the variables to unit variance is often performed prior to PLS modelling, in order to give all variables equal weight. However, in doing so, information on quantitative composition of the fractions would be discarded, which, however, turned out to be relevant in the present case since the unscaled data yielded a better model than after scaling. The resulting PLS model was validated with full cross validation (leave-one-out cross validation). It consisted of two significant PLS components and showed the statistical parameters summarized in Section 2.2 and Figure 2.

### 3.6. Biological Testing

In vitro assays for the bioactivity against *Trypanosoma brucei rhodesiense* (*Tbr*, bloodstream-form trypomastigotes, STIB 900 strain) and cytotoxicity tests against mammalian cells (L6-cell-line from rat skeletal myoblasts) were performed at the Swiss Tropical and Public Health Institute (Swiss TPH, Basel, Switzerland), according to established protocols and as previously described [18].

## 4. Conclusions

The considerable antitrypanosomal activity of Sage tincture reported previously can be attributed, at least for a major part, to its main diterpene constituents, abietane-lactones of the rosmanol type. It will be of particular interest to investigate, in further studies, the role of purified rosmaquinone congeners in this bioactivity. The result that different classes of constituents (flavonoid, diterpenes) account for the high activity of different fractions may indicate that they contribute in an additive or over-additive way to the overall anti-Tbr-activity of *S. officinalis* L. Studies on the effect of combinations of such compounds are thus warranted.

## Figures and Tables

**Table 1:** Antitrypanosomal activity (growth inhibition, GI, in % as compared to solvent-treated control cultures) of fractions at fixed concentrations of 2 and 10 µg/mL.

	% GI	
Fraction ID	10 µg/mL	2 µg/mL
FR3	10.2	11.2
FR5	28.6	15.2
FR8	18.4	20.9
FR10	15.7	14.2
FR12	24.3	19.5
FR13	59.3	20.5
FR14	71.0	19.3
FR15	70.1	25.7
FR18	79.3	11.1
FR19	100.0	18.9
FR20	100.0	28.6
FR22	75.6	51.0
FR24	84.6	26.6
FR27	65.3	37.9
FR29	58.8	30.8
FR32	30.7	21.2
FR36	24.4	18.9

**Table 2:** Chromatographic/mass spectral characteristics (UHPLC/+ESI QqTOF MS) of peaks A–H (compare Figure 1 and Figure 2).

Peak	Compound#	tR (min)	*m/z*		Mol. Formula
			[M+H]^+^	[M+Na]^+^	
A	-	2.4	293.0273	315.0065	n.d. ^1^
B	**1**	5.5	315.0864	337.0682	C_17_H_14_O_6_
C	**2**	5.9	347.1858	n.dt. ^2^	C_20_H_26_O_5_
D	**5**	7.0	361.2020	n.dt. ^2^	C_21_H_28_O_5_
E	**6**	7.5	375.2183	397.1982	C_22_H_30_O_5_
F	**8**	8.4	347.2205	n.dt. ^2^	C_21_H_30_O_4_
G	n.i. ^4^	8.7	283.1702	305.1518	C_19_H_22_O_2_
H	**9**	10.1	273.2578 ^3^	n.dt. ^2^	C_20_H_34_O

^1^ n.d.: not determined. ^2^ n.dt.: not detected. ^3^ [M+H−H_2_O]^+^. ^4^ n.i.: not identified, isomer of miltirone.

**Table 3:** Antitrypanosomal activity and cytotoxicity of the isolated compounds from Sage tincture. Data represent means of two independent IC_50_ determinations ± deviation from the mean. SI is the selectivity index = IC_50_(cytotox)/IC_50_(Tbr).

	IC_50_ *Tbr* (STIB 900)		IC_50_ Cytotox L6		SI
Compound	µg/mL	µM	µg/mL	µM	
Cirsimaritin (**1**)	1.7 ± 0.5	5.4	4.6 ± 0.37	15	2.8
Epirosmanol (**2**)	3.2 ± 0.3	9.3	5.0 ± 0.9	15	1.6
Rosmanol (**3**) + Rosmaquinone (**4**) (4:1)	1.1 ± 0.3	≈3.1	5.2 ± 0.0	15	≈4.9
7-*O*-Methylrosmanol (**5**)	8.3 ± 0.5	23	6.6 ± 0.8	18	0.8
7-*O*-Ethylrosmanol (**6**) + 7-*O*-Ethyl- rosmaquinone (**7**) + unidentified compound (6:4:1)	1.0 ± 0.43	≈ 2.7	17 ± 0.9	≈ 45	17
12-*O*-Methylcarnosic acid (**8**)	15 ± 2.3	44	20 ± 0	56	1.3
Manool (**9**)	2.6 ± 0.7	9.1	5.6 ± 0.5	19	2.1
Melarsoprol	0.0025 ± 0.005	0.0063			
Podophyllotoxin			0.009 ± 0.001	0.022	

## Supplementary Materials

The following are available online. Figure S1: UHPLC/+ESI QqTOF MSMS analysis of the investigated Sage tincture. Figures S2–S11: The 1H and 13C NMR spectra of compounds 2, 3+4, 5, 6+7, and 9. Table S1: Peaks detected in Sage tincture and results of their dereplication. Tables S2–S5: The 1H- and 13C NMR data of compounds **2**, **3**+**4**, **5**, **6**+**7,** and **9**.

## Sample Availability

Samples of the compounds **2**, **3**+**4**, **5**, **6**+**7,** and **9** are available from the authors.
