# Supplementary material for: Salvia officinalis L.: Antitrypanosomal Activity and Active Constituents against Trypanosoma brucei rhodesiense
Source: Molecules. 2021 May 27;26(11):3226. doi: 10.3390/molecules26113226 (PMC8199030; doi:10.3390/molecules26113226)
Supplement: Supplementary file 1 [file molecules-26-03226-s001.zip › molecules-1227827-supplementary.pdf]

Supplementary Material

Salvia officinalis: Antitrypanosomal Activity and Active Constituents against *Trypanosoma brucei rhodesiense*.

Núria Llurba Montesino [1], Marcel Kaiser [2,3], Pascal Mäser [2,3] and Thomas J. Schmidt [1,*]

[1] Institute of Pharmaceutical Biology and Phytochemistry (IPBP), University of Münster, PharmaCampus, Corrensstr. 48, D-48149 Münster, Germany; dagmar.flittner@web.de (D.F.)

[2] Swiss Tropical and Public Health Institute (Swiss TPH), Socinstrasse 57, CH-4051 Basel, Switzerland; marcel.kaiser@unibas.ch (M.K.); pascal.maeser@swisstph.ch (P.M.)

[3] University of Basel, Petersplatz 1, CH-4003 Basel, Switzerland

* Correspondence: thomschm@uni-muenster.de; Tel.: +49-251-83-33378 (T.J.S.)

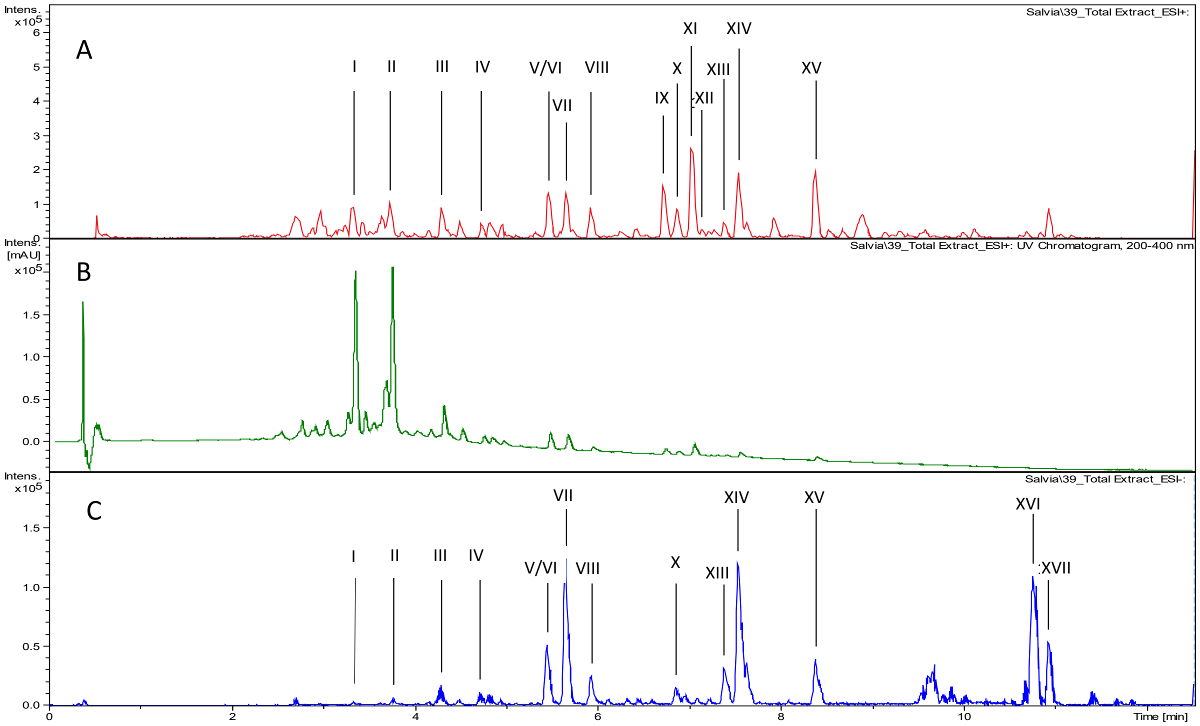

Figure S1. UHPLC/+ESI QqTOF MSMS analysis of the investigated Sage tincture. A: +ESI base peak ion chromatogram (m/z 100–1000). B: UV Chromatogram 200–400 nm. C: -ESI base peak ion chromatogram (m/z 100–1000).

Table S1. Peaks detected in Sage tincture (compare Figure S1) by UHPLC/ESI QqTOF MSMS and results of their dereplication.

Peak	tR	Ion [M+H]$^+$	Ion [M-H]$^-$	Main Fragment(s) (ESI+)	UV ◎ max	Suggested compound
I	3.3	463.0897	461.4708	287.0566	204, 272, 336	luteolin-7-glucuronide
II	3.7	361.0900	359.4232	163.0386	288, 328	rosmarinic acid
III	4.3	287.0573	285.3410	n.d.	236, 289, 325	luteolin
IV	4.7	271.0610	269.3371	n.d.	240, 286, 325	apigenin
V	5.5	315.0864	313.3915	n.d.	240, 281,333	cirsimartin
VI	5.6	347.1838	345.5095	301.1802	n.d.	rosmanol or isomer
VII	5.7	347.1847	345.5111	301.1801/ 329.1745	240, 286, 332	rosmanol or isomer
VIII	5.9	347.1879	345.5095	301.1971/ 329.1742	244, 288, 326	rosmanol or isomer
IX	6.7	329.1018	n.d.	n.d.	244, 283,328	salvigenin
X	6.9	361.2003	343.4937	329.1746/ 301.1785	244,290	7-O-methylrosmanol or isomer
XI	7.0	361.2010	n.d.	329.1748/ 301.1790	244, 288	7-O-methylrosmanol or isomer
XII	7.1	331.1898	n.d.	285.1836	248	carnosol
XIII	7.4	375.2183	373.5550	329.1763/ 301.1798	248, 290, 322	7-O-ethylrosmanol or isomer
XIV	7.5	375.2163	373.5568	329.1760/ 301.1808	248, 288, 325	7-O-ethylrosmanol or isomer
XV	8.4	347.2005	345.5459	301.2163		12-O-methyl-carnosic acid
XVI	10.8	n.d.	535.8133	n.d.	n.d.	n.i.
XVII	10.9	n.d.	549.8360	n.d.	n.d.	n.i.

Abbreviations: tR, retention time; n.d., not detected; n.i., not identified.

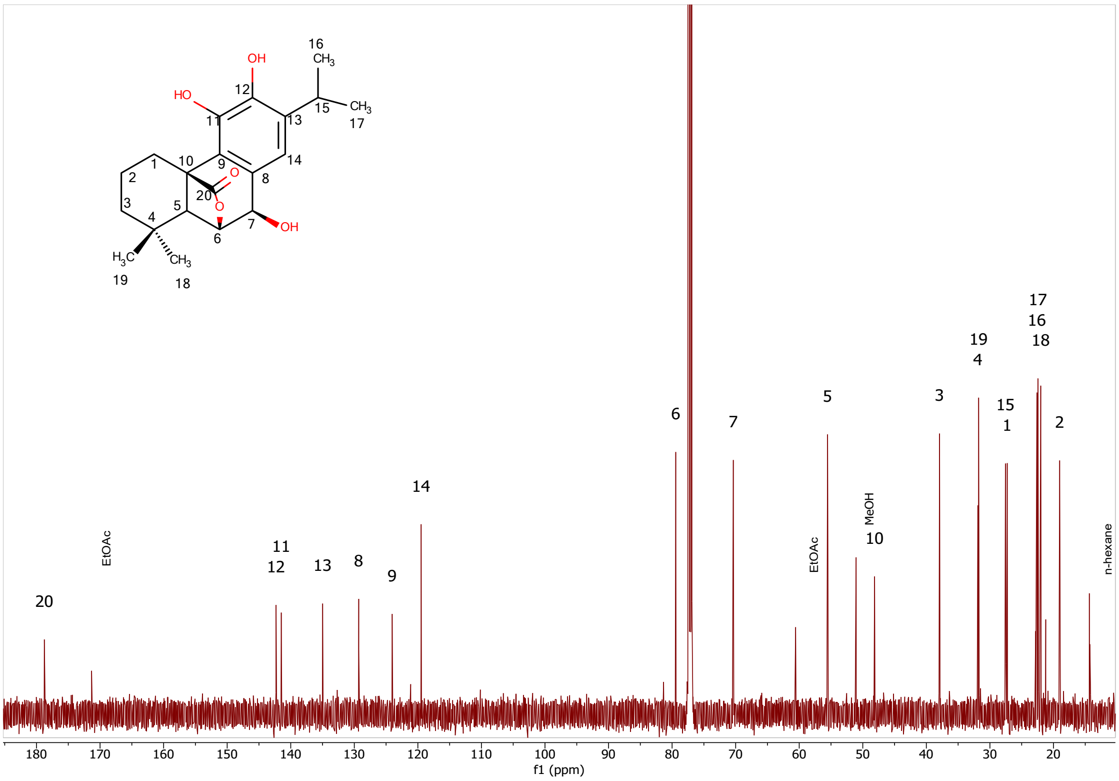

Figure S2. ^{13}C NMR spectrum of compound **2** (150 MHz, $CDCl_3$).

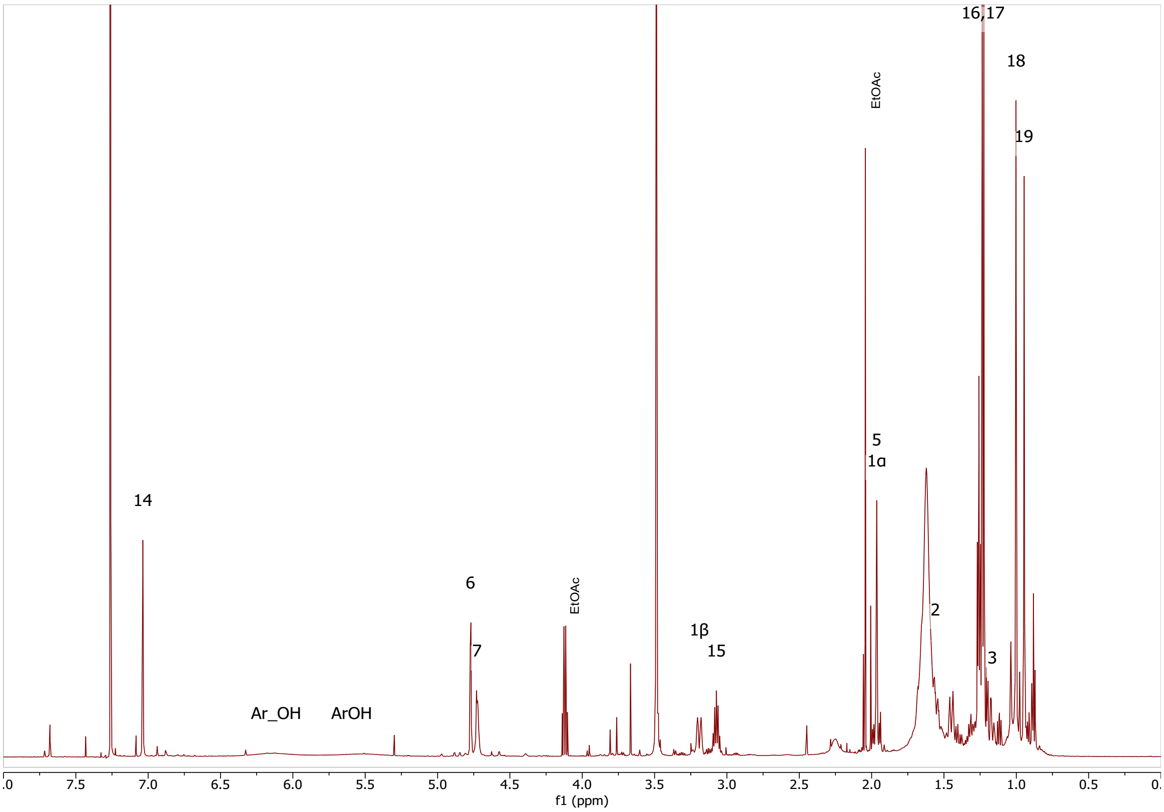

Figure S3. ^{1}H NMR spectrum of compound **2** (600 MHz, $CDCl_3$).

Table S2. ^{1}H- and ^{13}C-NMR data ($CDCl_3$, 600 and 150 MHz, respectively) of compound **2** compared with literature [6].

	Epirosmanol **(2)**		
	δ [1]H *mult (J* in Hz*)*	δ [1]H *mult (J* in Hz*) [6]*[a]	δ [13]C
1 α	1.96 *dt (14.2/ 5.5*)	-	27.27
1 β	3.19 *br d (14.2)*	3.19 *br d (n.d)*	
2 α	1.67 *m*	-	19.03
2 β	1.55 *dt (13.9/ 3.6)*	-	
3 α	1.17 *dd (13.5/ 3.3)*	-	37.92
3 β	1.45 *dt (13.5/3.3)*	-	
4	-	-	31.89
5	1.96 *s*	1.97 *s*	55.54
6	4.78 *d (3.2)*	4.77 *overlapping signals*	79.43
7	4.73 *br d (3.6)*	-	70.37
8	-	-	129.30
9	-	-	124.04
10	-	-	48.14
11	-	-	141.49
12	-	-	142.31
13	-	-	134.98
14	7.04 *s*	7.04 *s*	119.48
15	3.07 *sept (6.8)*	3.08 *sept* (7.0)	27.56
Me-16	1.23 *d (6.7)*	1.23 *d (7)*	22.60
Me-17	1.24 *d (6.7)*	-	22.45
Me-18	1.00 *s*	1.00 *s*	21.93
Me-19	0.95 *s*	0.95 *s*	31.77
20	-	-	178.77
Ar-OH	5.50 *br s*	5.50 *br s*	-
Ar-OH	6.12 *br s*	6.18 *br s*	-

[a](300 MHz $CDCl_3$).

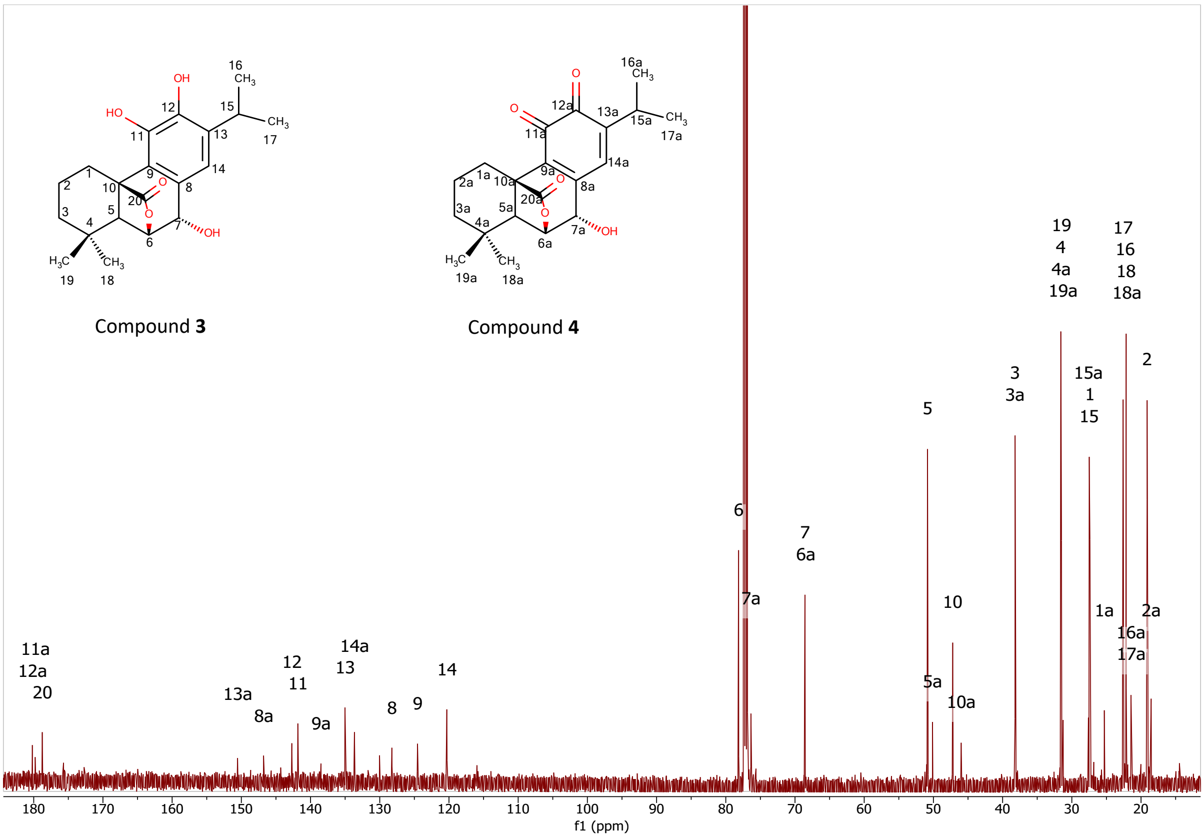

Figure S4. ^{13}C NMR spectrum of compounds **3+4** (150 MHz, $CDCl_3$).

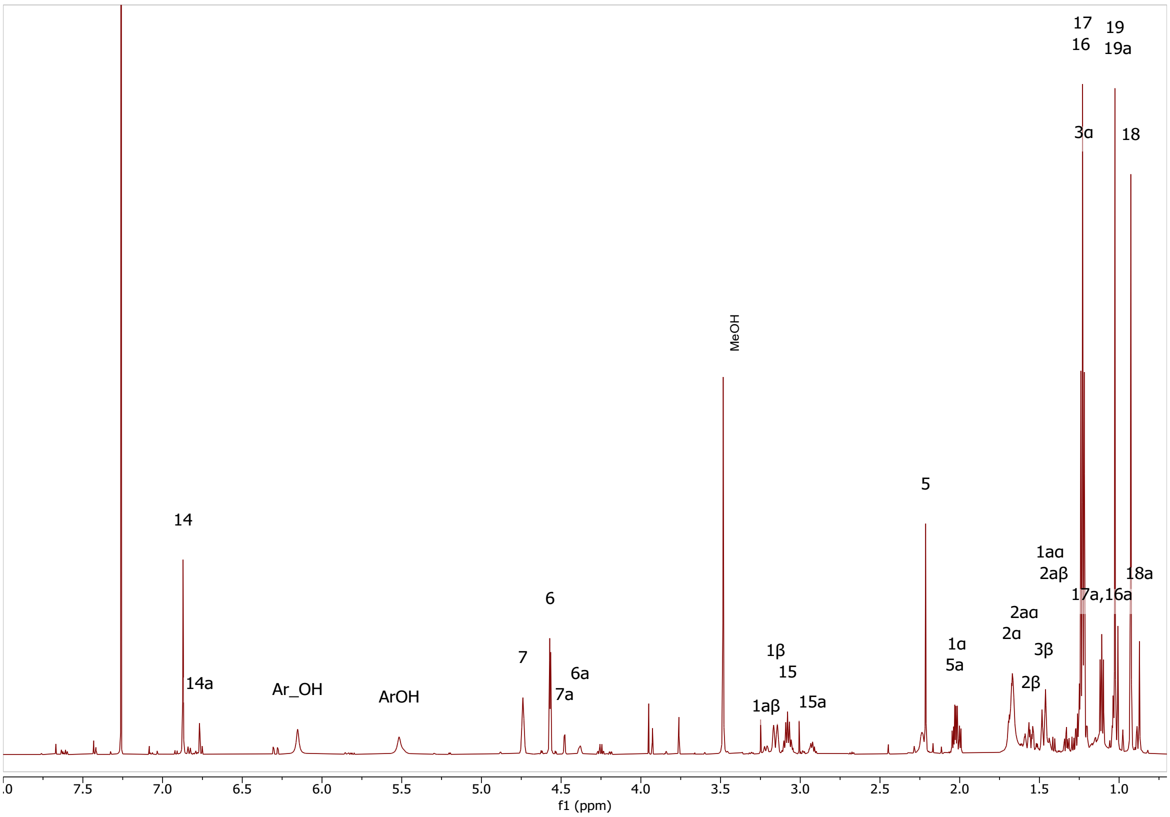

Figure S5. ^{1}H NMR spectrum of compounds **3+4** (600 MHz, $CDCl_3$).

Table S3. ^{1}H-NMR and ^{13}C-NMR data ($CDCl_3$, 600 and 150 MHz, respectively) of compounds **3** and **4** compared with literature [6, 7].

	Rosmanol (**3**)*			*Rosmaquinone* (**4**)			
	δ ^{1}H *mult (J* in Hz)	δ ^{1}H *mult (J* in Hz)	δ ^{13}C	δ ^{1}H *mult (J* in Hz)	δ ^{1}H *mult (J* in Hz) [7][b]	δ ^{13}C	δ ^{13}C[b]
1 α	2.01 *dt (14.2/5.3)*	2.00 *dt*	27.47	1.44	1.45	25.4	25.0
1 β	3.20 *br d (14.3)*	3.21 *br d*		3.21 *br d*	3.21 *br d* (10.5)		
2 α	1.66 <u>*m*</u>	-	19.20	1.59	1.60 *m*	18.6	18.2
2 β	1.54 *dt (13.7/*	-		1.46	1.45		
3 α	1.22 *d (5.7)*	-	38.32	n.d.	1.22 *m*	38.4	37.8
3 β	1.47 *dt (13.4/*	-		n.d.	1.45		
4	-	-	31.53	-	-	31.2	31.2
5	2.22 *s*	2.21 *s*	50.83	2.03s	2.05 *s*	50.75	49.8
6	4.56 *d (3.4)*	4.57 *d (3.3)*	78.16	4.38 *br s*	4.52 *d (3.5)*	76.41	76.1
7	4.73 *d (3.4)*	4.74 *d (3.3)*	68.62	4.48 *d (3.1)*	4.40 *d (3.5)*	68.49	68.0
8	-	-	128.30	-	-	146.8	146.7
9	-	-	124.48	-	-	138.51	138.1
10	-	-	47.25	-	-	46.02	45.7
11	-	-	142.43	-	-	179.79	179.5
12	-	-	142.71	-	-	180.2	179.9
13	-	-	135.61	-	-	150.55	150.1
14	6.86 *s*	6.87 *s*	120.11	6.77 *s*	6.79 s	133.67	133.5
15	3.12 *sept (6.9)*	3.20 *sept (7.0)*	27.38	2.92	2.93 *hept* (7)	27.65	27.3
Me-16	1.21 *d (6.6)*	1.22 *d 7(.0)*	22.40	1.11	1.06 *d (7)*	21.5	21.1
Me-17	1.20 *d (6.6)*	1.23 *d (7.0)*	22.72	1.11	1.08 *d(7)*	21.46	21.1
Me-18	1.03 *s*	1.03 *s*	31.64	1.01 *s*	1.02 *s*	31.3	30.9
Me-19	0.93 *s*	0.93 *s*	22.22	0.87 *s*	0.88 *s*	22.05	21.7
20	-	-	178.78	-	-	-	-
Ar-OH	6.15 *br s*	-	-	-	-	-	-
ArOH	5.51 *br s*	-	-	-	-	-	-

* Compound **3** was present as the major component of a mixture (4:1) with its oxidized o-quinoid form, rosmaquinone **4**.

[a] (300 MHz $CDCl_3$)

[b] (^{1}H-NMR; 300 MHz $CDCl_3$, ^{13}C-NMR; 75 MHz $CDCl_3$)

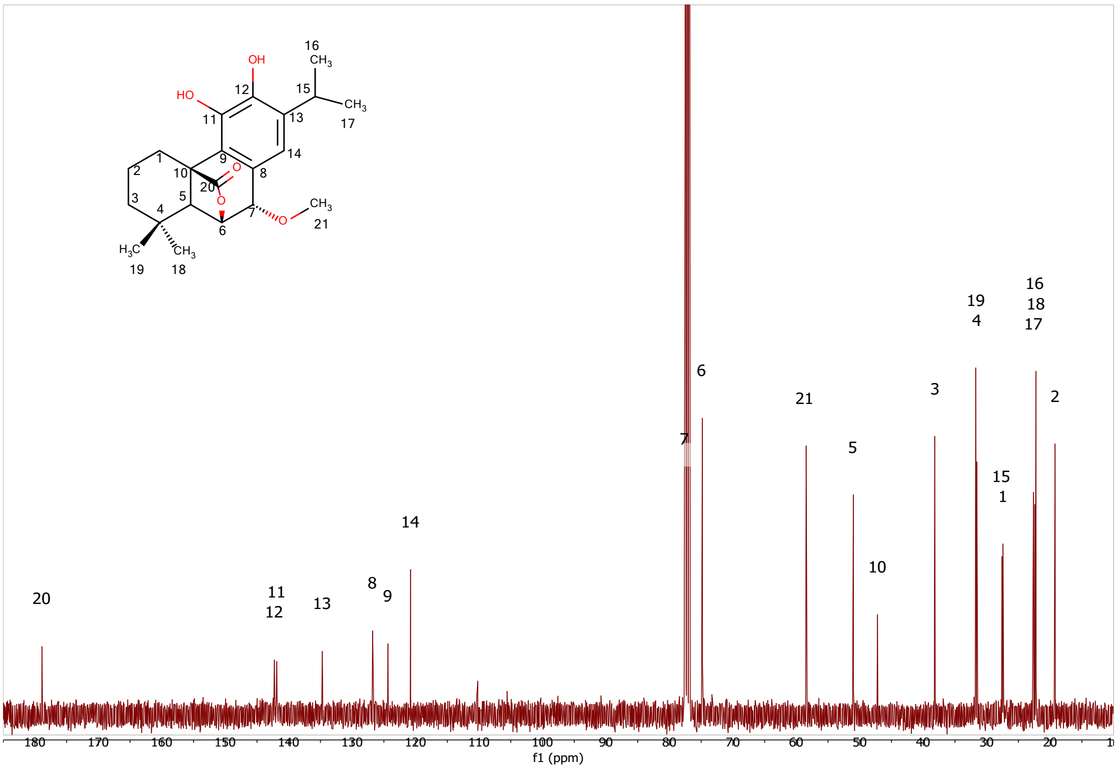

Figure S6. ^{13}C NMR spectrum of compound **5** (150 MHz, $CDCl_3$).

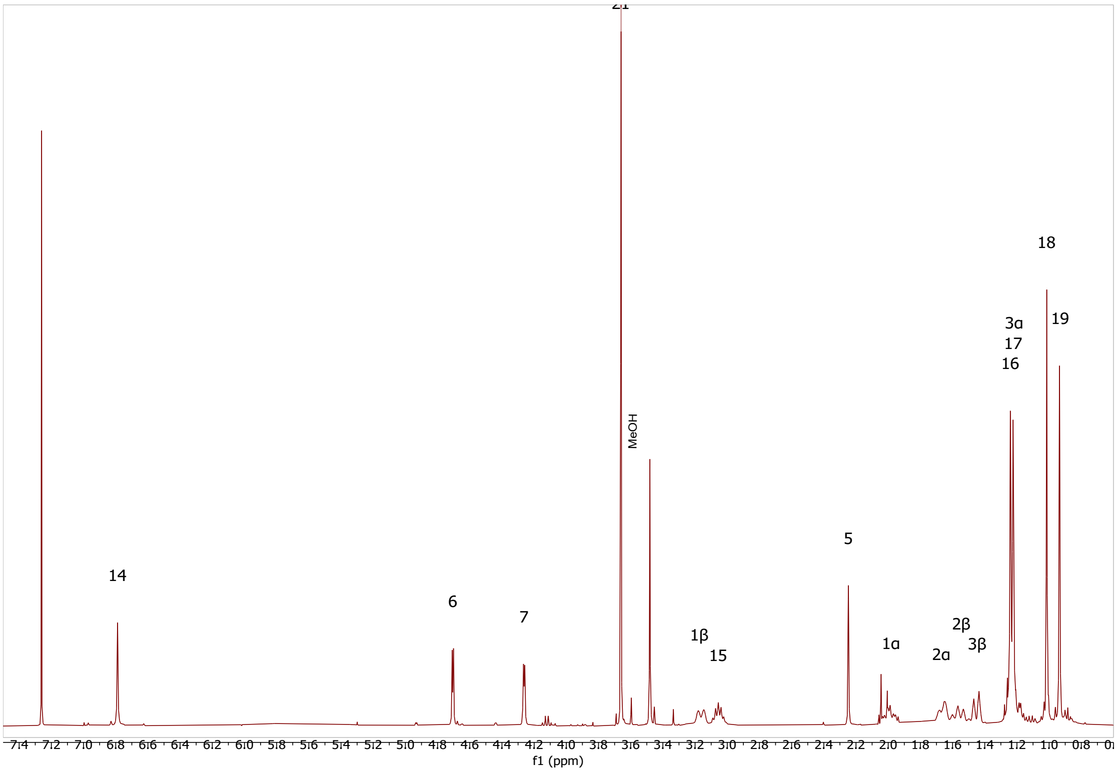

Figure S7. ^{1}H NMR spectrum of compound **5** (600 MHz, $CDCl_3$).

Table S4. ^{1}H-NMR and ^{13}C-NMR data ($CDCl_3$, 600 and 150 MHz, respectively) of compound **5** compared with literature [9].

	7-O-methylrosmanol (**5**)			
	δ ^{1}H *mult* (*J* in Hz)	δ ^{1}H *mult* (*J* in Hz) [9][a]	δ ^{13}C	δ ^{13}C [9][a]
1 α	1.99 *td (13.8/5.4)*	1.96 *td (13.8/ 4.8)*	27.37	27.3
1 β	3.16 *br d (13.8)*	3.26 *d (13.8)*		
2α	1.66 *dd (13.5/3.9)*	1.47 *br q* (13.8)	19.17	18.9
2β	1.54 *br dt (13.5/3.9)*	1.59-1.68 *m*		
3α	1.22 *m*	1.22 *m*	38.09	38.0
3β	1.45 *dt (13.2/ 3.4)*	1.44 *br d (13.2)*		
4	-	-	31.52	31.5
5	2.25 *s*	2.23 *s*	51.07	50.9
6	4.70 *d (3.10)*	4.70 *d* (3)	74.89	77.5
7 β	4.26 *d (3.1)*	4.25 *d (3)*	77.66	74.7
8	-	-	126.36	126.5
9	-	-	124.34	124.1
10	-	-	47.17	47.0
11	-	-	141.86	142.4
12	-	-	142.22	141.9
13	-	-	134.69	134.9
14	6.78 *s*	6.77 *s*	120.78	120
15	3.05 *sept (6.9)*	3.05 *sept (7.2)*	27.66	27.1
16	1.24 *d (6.9)*	1.18 *d (6.6)*	22.37	22.4
17	1.22 *d (6.8)*	1.18 *d (6.6)*	22.58	22.1
Me-18	1.01 *s*	1.00 *s*	31.65	31.3
Me-19	0.93 *s*	0.90 *s*	22.19	21.9
20	-	-	178.89	179.2
21 $O-CH_3$	3.66 *s*	3.65 *s*	58.65	58.3
Ar-OH	n.d.	6.00 *s*	-	-
Ar-OH	n.d.	6.00 *s*	-	-

[a]^{1}H-NMR; 600 MHz $CDCl_3$; ^{13}C-NMR: 150MHz $CDCl_3$)

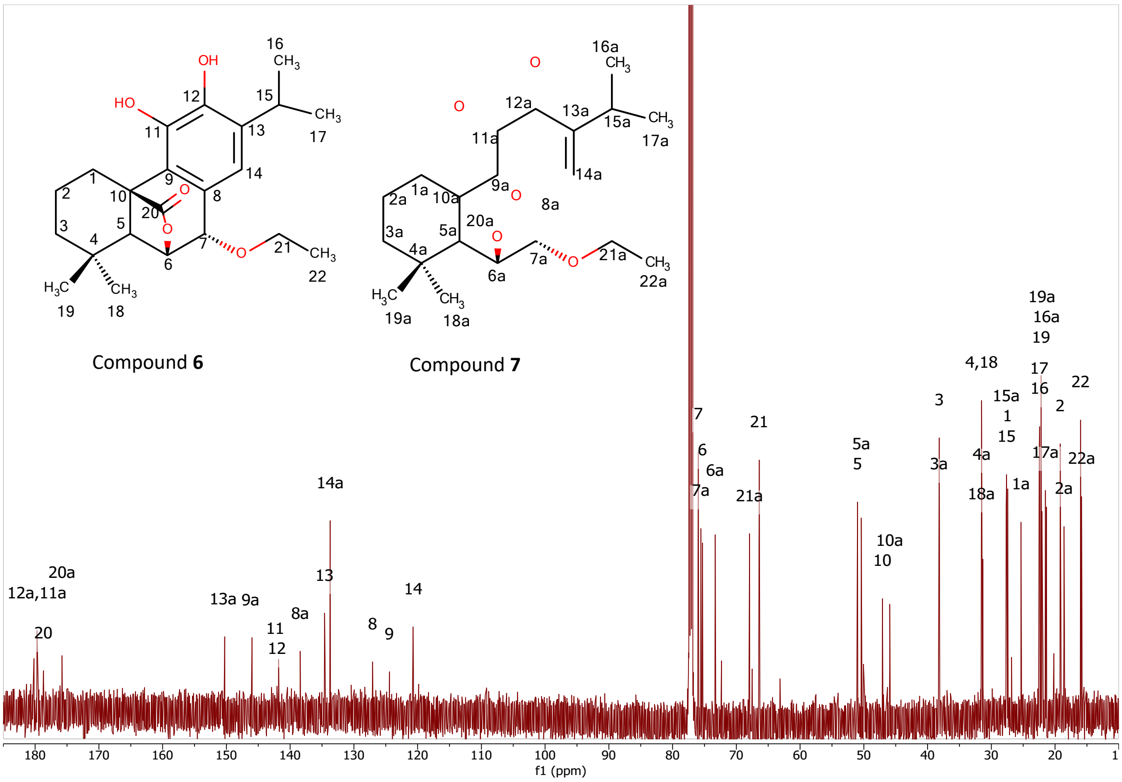

Figure S8. ^{13}C NMR spectrum of compounds **6** + **7** (150 MHz, $CDCl_3$).

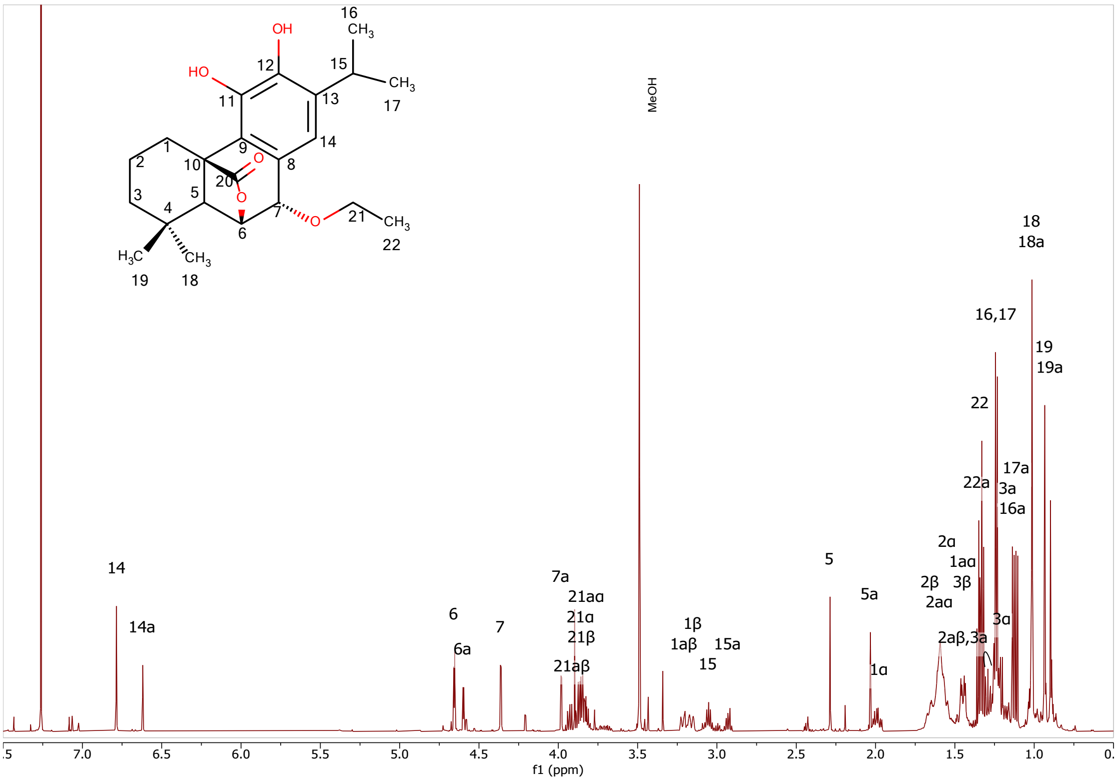

Figure S9. ^{1}H NMR spectrum of compounds **6** + **7** (600 MHz, $CDCl_3$).

Table S5. ^{1}H-NMR and ^{13}C-NMR data ($CDCl_3$, 600 and 150 MHz, respectively) of compound **4** (major compound) and compound **5** (minor compound) (mixture 6:4:1 7-O-ethylrosmanol, 7-O-ethylrosmaquinone: non identified compound). Data of **4** are compared with literature [11], those of **5** are reported for the first time.

	7-O-ethylrosmanol (**4**)				*7-O-ethylrosmaquinone* (**5**)	
	δ ^{1}H *mult* (*J* in Hz)	δ ^{1}H *mult* (*J* in Hz)[11][a]	δ ^{13}C	δ ^{13}C [11] [a]	δ ^{1}H *mult* (*J* in Hz)	δ ^{13}C
1α	1.99 *dt (14.1/5.8)*	1.99 *dd (5.1/13.8)*	27.43	27.2	1.45 *dd*	25.34
1β	3.16 *br d (14.2)*	3.19 *br d (13.8)*			3.21 *br d*	
2α	1.55 *dt (14.5/3.7)*	1.54 *dt (3.2/13.4)*	19.19	19.0	1.59 *indef*	18.59
2β	1.66 *m*	1.69 *br d (13.4)*			1.46	
3α	1.21 *d (6.9)*	1.19 *m*	38.17	38.0	1.21 (6.7)	38.2
3β	1.45 *m*	1.45 *br d 13)*			1.45	
4	-	-	31.53	31.4	-	31.53
5	2.29 *s*	2.29 *s*	50.99	50.9	2.03 *s*	50.40
6	4.66 *d (3.2)*	4.68 *d (3.1)*	75.31	75.3	4.60 *d (3.1)*	73.32
7	4.36 *d (3.2)*	4.36 *d (3.1)*	75.97	75.8	3.98 *d (3.1)*	75.57
8	-	-	127.11	126.9	-	138.42
9	-	-	124.44	124.2	-	145.98
10	-	-	47.08	47.0	-	45.94
11	-	-	141.80	142.0	-	179.71
12	-	-	142.09	142.0	-	180.19
13	-	-	134.6	134.9	-	150.26
14	6.79 *s*	6,77 *s*	120.5	120.5	6.62 *d (1.2)*	133.71
15	3.05 *sept (7.1)*	3.07 *sept (6.7)*	27.61	27.3	2.93 *h (1.2/7)*	27.67
16	1.24 *d (6.9)*	1.21 *d (6.7)*	22.42	22.2	1.13 *d (6.9)*	21.35
17	1.24 *d (6.9)*	1.23 *d (6.7)*	22.49	22.4	1.11 *d (6.9)*	21.51
Me-18	1.01 *s*	1.02 *s*	31.50	31.4	1.01 *s*	31.57
Me-19	0.93 *s*	0.94 *s*	22.18	22.0	0.90 *s*	22.06
20	-	-	178.71	179.0	1.13 *d*	175.79
21 O-CH_2	-	-	66.41	66.2	3.82 *m*	67.93
21 O-CH_2	3.86 *dd*	3.85 *q* (7.0)	66.41	66.2	3.92 *ddd 9.9/7.4/ 2.8)*	67.93
Me-22	1.34 *t (6.8)*	1.35 *t* (7)	15.99	15.8	1.36 *t (7.05)*	15.84
Ar-OH	n.d.	6.06 *br s*	-	-	-	-
Ar-OH	n.d.	5.68 *br s*	-	-	-	-

[a] ^{1}H-NMR; 400 MHz $CDCl_3$; ^{13}C-NMR 100MHz $CDCl_3$

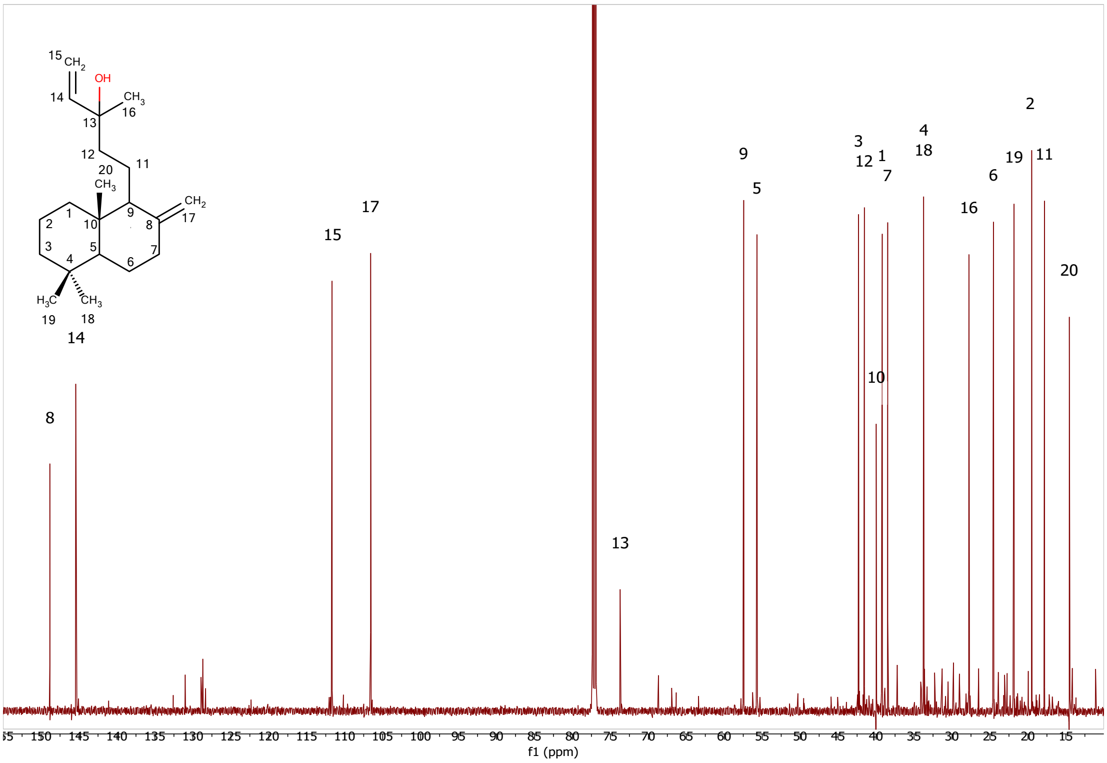

Figure S10. ^{13}C NMR spectrum of compound **9** (150 MHz, $CDCl_3$).

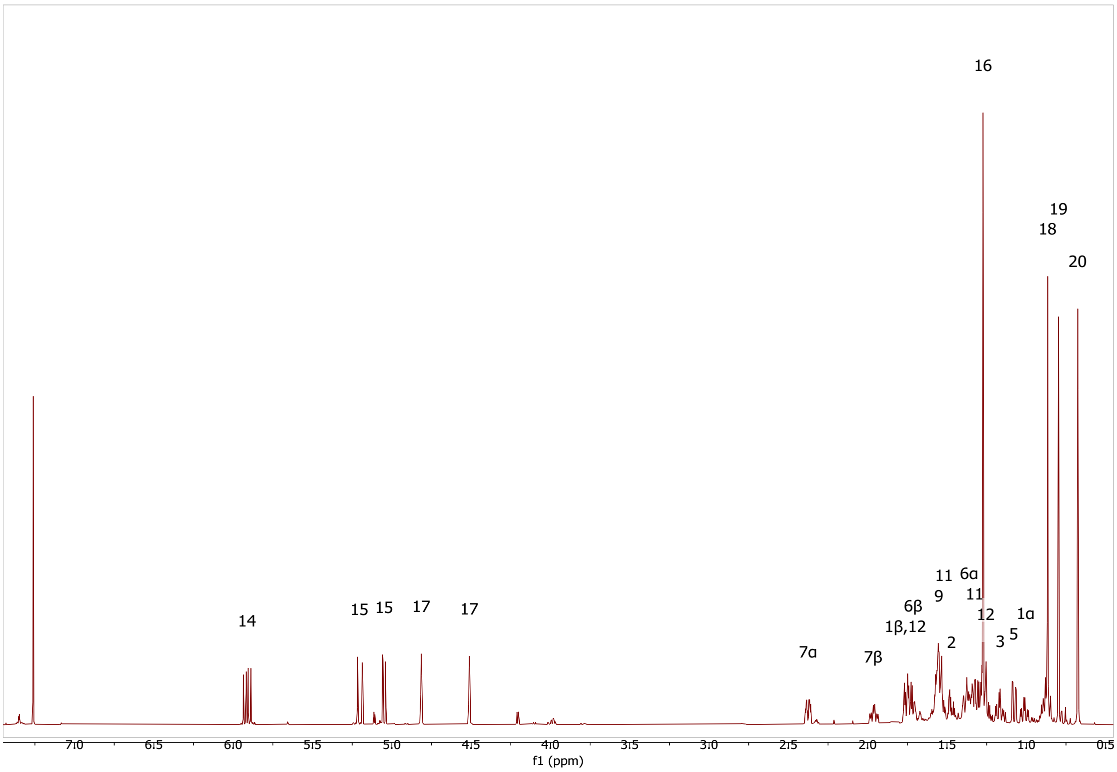

Figure S11. ^{1}H NMR spectrum of compound **9** (600 MHz, $CDCl_3$).

Table S6. ^{1}H- and ^{13}C-NMR data ($CDCl_3$, 600 and 150 MHz, respectively) of compound **9** compared with literature [15, 16].

	Manool (**9**)			
	δ ^{1}H *mult (J* in Hz*)*	δ ^{1}H *mult (J* in Hz*)* *[16]* [a]	δ ^{13}C	δ ^{13}C [15][b]
1α	1.01 *td (13.2/4.2)*		39.23	39.0
1β	1.75 *m*			
2α	1.56 *m*		19.55	19.0
2β	1.47 *d*t (13.9/3.6)			
3α	1.38 *m*		42.36	42.1
3β	1.17 *td (13.5/4.0)*			
4	-		33.73	33.5
5	1.07 *dd(12.6/2.8)*		55.73	55.5
6α	1.31 *dd (4.5/13.2)*		24.57	24.4
6β	1.70 *m*			
7α	2.37 *dq*		38.50	38.3
7β	1.97 *td (12.9/5.2)*			
8	-		148.85	148.4
9	1.54 *m*		57.47	57.2
10	-		40.06	39.8
11	1.53 *m*		17.86	17.6
	1.35 *m*			
12	1.25 *q* (4.6/1.7)		41.58	41.3
	1.74 *dd (1.4/4.2)*			
13	-		73.78	73.4
14	5.91 *dd (17.4/10.8)*	5.92 *dd (11/ 17.5*)	145.47	144.9
15	5.05 *dd (10.8/1.2)*	5.05 *n.d. (2, 11)*	111.68	111.4
	5.20 *dd (17.4/1.3)*	5.21 *dd (2, 17.5)*		
Me- 16	1.27 *s*	1,26 *s*	27.82	27.9
17	4.81 *q (3.2/1.6)*	4.8 *br s*	106.57	106.2
	4.51 *d (3.12/1.4)*	4.47 *br s*		
Me-18	0.86 *s*	0.87 *s*	33.78	33.5
Me-19	0.79 *s*	0.78 *s*	21.87	21.7
Me- 20	0.67 *s*	0.6 *s*	14.58	14.4

[a] 200 MHz $CDCl_3$; [b]300 MHz $CDCl_3$
